# Dynamical state transitions into addictive behaviour and their early-warning signals

**DOI:** 10.1098/rspb.2017.0882

**Published:** 2017-08-02

**Authors:** Jerome Clifford Foo, Hamid Reza Noori, Ikuhiro Yamaguchi, Valentina Vengeliene, Alejandro Cosa-Linan, Toru Nakamura, Kenji Morita, Rainer Spanagel, Yoshiharu Yamamoto

**Affiliations:** 1 Department of Physical and Health Education, Graduate School of Education, The University of Tokyo, Hongo 7-3-1, Bunkyo-ku, 113-0033 Tokyo, Japan; 2 Institute of Psychopharmacology, Central Institute of Mental Health, Medical Faculty Mannheim, Heidelberg University, J 5, 68159 Mannheim, Germany; 3 Department of Genetic Epidemiology in Psychiatry, Central Institute of Mental Health, Medical Faculty Mannheim, Heidelberg University, J 5, 68159 Mannheim, Germany; 4 Neuronal Convergence Group, Max Planck Institute for Biological Cybernetics, Spemannstrasse 38, 72076, Tuebingen, Germany

**Keywords:** critical transition, early-warning signals, locomotor activity, drinking behaviour, alcoholism

## Abstract

The theory of critical transitions in complex systems (ecosystems, climate, etc.), and especially its ability to predict abrupt changes by early-warning signals based on analysis of fluctuations close to tipping points, is seen as a promising avenue to study disease dynamics. However, the biomedical field still lacks a clear demonstration of this concept. Here, we used a well-established animal model in which initial alcohol exposure followed by deprivation and subsequent reintroduction of alcohol induces excessive alcohol drinking as an example of disease onset. Intensive longitudinal data (ILD) of rat drinking behaviour and locomotor activity were acquired by a fully automated drinkometer device over 14 weeks. Dynamical characteristics of ILD were extracted using a multi-scale computational approach. Our analysis shows a transition into addictive behaviour preceded by early-warning signals such as instability of drinking patterns and locomotor circadian rhythms, and a resultant increase in low frequency, ultradian rhythms during the first week of deprivation. We find evidence that during prolonged deprivation, a critical transition takes place pushing the system to excessive alcohol consumption. This study provides an adaptable framework for processing ILD from clinical studies and for examining disease dynamics and early-warning signals in the biomedical field.

## 1. Background

The recent surge in the development of wearable and mobile biomedical sensing technology has made it possible to acquire long-term continuous biomedical signals, also known as biomedical intensive longitudinal data (ILD) [1]. These data will be increasingly used in healthcare applications with information and communications technologies (ICT) and will contribute in particular to the understanding of disease dynamics [2,3]. Analysis of this type of data has the potential to predict disease onset and thus may pave the way for the development of adaptive prevention and treatment strategies [4].

Biomedical ILD reflect the underlying nonlinear, multi-scale dynamics of complex biological systems operating on multiple time scales ranging from, for example, minutes or hours or days or weeks [2]. Critical transitions, where a system shifts abruptly from one stable state to another, are one of the most commonly observed features of complex systems with nonlinear behaviour [5,6] and may be preceded by various early-warning signals such as the so-called critical slowing down (increased low-frequency, auto-correlated variability near transition points) [5,6]. Theoretical studies and computational simulations have yielded evidence for critical transitions in natural phenomena, but only a few biological and medical studies have examined the validity of these concepts in medical applications (see [3] for a review). Furthermore, dynamical aspects of disease onset across transition (bifurcation) points have not been directly explored, as until now it has been difficult to contiguously and continuously monitor disease dynamics in standard clinical settings. Here, we provide an example of state transitions in the biomedical field by studying disease dynamics in a well-established animal model of alcohol addiction.

Alcohol consumption patterns in people at risk for alcohol-use disorder or even in alcohol addicted patients are characterized by repeated abstinence periods followed by relapse and excessive alcohol consumption. Early abstinence refers to acute withdrawal, whereas protracted abstinence refers to an extended period where overt withdrawal signs are no longer seen [7–9]. On the molecular level and also on the neuronal network level, critical changes occur during abstinence phases and it is suggested that those changes contribute to the development of addictive behaviour [8,10]. Abstinence—be it early or protracted—can be modelled in animals by introducing a deprivation period. The alcohol deprivation effect (ADE) model is a well-established animal model of relapse-like excessive alcohol drinking in which rats are given alcohol for a baseline period followed by a deprivation period and subsequent reintroduction of alcohol [11,12]. Following reintroduction of alcohol, excessive alcohol consumption is seen for several days reflecting relapse-like drinking behaviour, after which sustained increased alcohol consumption is observed. Numerous studies have used the ADE model to study behavioural, neurobiological and pharmacological aspects underlying the transition to excessive alcohol consumption [11–14].

In this study, we used the ADE model and a novel drinkometer system [15], a computer-based system enabling ILD collection for rodent drinking behaviour and locomotor activity, to study dynamical state transitions characterizing disease onset. To this end, we examined the statistical and dynamical properties of drinking behaviour and locomotor activity ILD across baseline, deprivation and reintroduction phases, showing that both underwent dynamical state transitions from a stable baseline state to a new stable state with preference for stronger alcohol and increased consumption, with distinct early-warning signals during the early deprivation period. Importantly, we observe that although the deprivation pushes the system to its tipping point, the transition itself takes place during prolonged deprivation (i.e. protracted abstinence) spontaneously without further manipulation.

## 2. Material and methods

### (a) Animals

Twenty-four two-month-old male Wistar rats (from the breeding colony at the CIMH, Mannheim, Germany) were subjected to the ADE paradigm. All animals were housed individually in standard rat cages (Eurostandard Type III; Ehret, Emmendingen, Germany) under an artificial 12 L : 12 D cycle (lights on at 7.00). Room temperature was kept constant (temperature: 23 ± 1°C). Standard laboratory rat food (Ssniff, Soest, Germany) and tap water were provided ad libitum throughout the experiment. Body weights were measured weekly.

### (b) Experimental paradigm

The ADE paradigm [8–15] is a tightly controlled experimental procedure to model excessive relapse-like drinking in rodents. The procedure involves a long-term baseline period of voluntary alcohol consumption in a four-bottle free-choice paradigm with water and three different concentrations of ethanol (5, 10 and 20%, reflecting human consumption of beer, wine and spirits), followed by subsequent deprivation and reintroduction phases. When deprived of alcohol after long-term (i.e. two months) voluntary consumption, rats show withdrawal symptoms during early deprivation [9,11]. While acute deprivation (i.e. for at least 3 days) can trigger a transient ADE, it has been shown that longer periods of deprivation (greater than one week) result in longer lasting behavioural and neurobiological adaptations consistent with the development of addictive behaviour. The reintroduction phase following deprivation is thus reminiscent of ‘relapse’-like behaviour, characterized by increases in alcohol intake and preference [8–15].

In this study, the ADE paradigm was applied while a drinkometer system acquired drinking behaviour and locomotor activity ILD sets from single animals in 1 min bins continuously over several months. After two weeks of habituation to the animal room, rats were given ad libitum access to tap water and 5, 10 and 20% ethanol solutions, and data recording was started to monitor baseline drinking activity. Animals were exposed to alcohol for a baseline period of eight weeks; behaviour had stabilized by the last week of the baseline (denoted as BASE). The baseline period was followed by a two-week deprivation phase in which all bottles were filled with tap water (deprivation (DEP: DEP_wk1_, DEP_wk2_)), and four weeks of measurements following ethanol reintroduction (ER for four weeks: ER_wk1_, ER_wk2_, ER_wk3_, ER_wk4_) (electronic supplementary material, figure S1*a*). Electronic supplementary material, figure S1*b* illustrates a sample ILD set for drinking behaviour and locomotor activity. Hereafter, we refer to BASE, DEP_wk1_, DEP_wk2_, ER_wk1_, ER_wk2_, ER_wk3_ and ER_wk4_ as the ‘experimental periods'.

### (c) High-resolution recording of alcohol drinking behaviour

The drinkometer system is a novel method to monitor drinking behaviour in rodents [15] and was developed in collaboration with TSE Systems (Bad Homburg, Germany). It enables long-term high-resolution continuous recording of liquid consumption by amount and time in a standard rat home-cage. The system is equipped with four drinking stations to allow liquid choice. The drinking station consists of a glass vessel containing the liquid and a high-precision sensor for weighing the amount of liquid removed from the glass vessel. In this study, the four drinking bottles contained tap water and 5, 10 and 20% ethanol solutions (v/v), which were prepared from 96% ethanol (Sigma-Aldrich, Taufkirchen, Germany) and diluted with tap water. Spillage and evaporation were minimized using special bottle caps. All drinking stations were monitored by a computer. The system features ultra-high-resolution recording capability—down to 0.01 g (however, values of 0.01 g are too small to be drinking events and were considered as measurement artefacts and were excluded from the analysis, see the electronic supplementary material). The whole system is mounted on a custom-made free-swinging steel frame to avoid any environmental disturbances. The drinkometer system measures the weight of a vessel in 200 ms steps and saves it in 1 s steps. The sampling interval was set at 1 min, giving per-minute values of solutions consumed.

### (d) High-resolution recording of locomotor activity

Home-cage spontaneous locomotor activity was monitored by the use of an infrared sensor placed above each cage (30 cm from the bottom) connected to a recording and data storing system (Mouse-E-Motion, Infra-e-motion, Henstedt-Ulzburg, Germany). The devices continuously sampled whether the rats were moving or not. The sensors detect (and count) body movements of rats of at least 1.5 cm from one sample point to a successive one. The detection of motion is based upon the warmth radiation in the infrared range emitted by the animals. The data measured by each Mouse-E-Motion device were downloaded into a personal computer and processed with Microsoft Excel/VBA and Matlab (R2012). The locomotor activity (i.e. number of movements) captured was recorded in 1 min intervals in both light and dark phases during the entire experiment. This resulted in a time series of number of movements per minute over the entire experiment, comprising the locomotor activity ILD set.

### (e) Data analysis

#### (i) Alcohol preference

Alcohol consumption was calculated during five periods: the last week before ethanol deprivation (BASE), and four weeks following ethanol reintroduction (ER_wk1_, ER_wk2_, ER_wk3_ and ER_wk4_) (bottles contained no ethanol during DEP_wk1_ and DEP_wk2_). Intake values were calculated in g kg d^−1^ for each solution and summed (accounting for the weight of the animal and molecular weight of ethanol and concentration of the ethanol solution). Alcohol preference (PF) was calculated for the same five periods by calculating density of alcohol consumed as follows:2.1
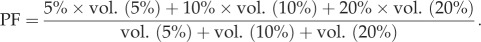


#### (ii) Transition matrices

For each animal, transitions between the four different bottles (water, 5, 10, 20%) were examined for all experimental periods. Any minutes in which drinking events occurred were counted; for minutes where multiple solutions were consumed, all transitions (stays and switches) were counted. For the full matrix, see the electronic supplementary material, table S1.

The probability to stay (stay ratio, *P*_stay_) was estimated for each animal by aggregating the total number of stays and switches of all alcoholic solutions:2.2
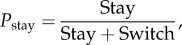
giving an index of the pattern of alcohol drinking behaviour. It should be noted that as water was included in the calculation of transition matrices, two drinking events from a single bottle separated by water (i.e. 5% → water → 5%) were not counted as ‘stays’.

#### (iii) Locomotor activity data

Rats were placed in new clean cages once a week, resulting in a corresponding locomotor artefact being observed. To account for this effect, the 24 h (from 00.00 to 00.00) within which each cage cleaning was done was removed from the analysis of locomotor data, resulting in 6 out of every 7 days of locomotor data being analysed. This resulted in a dataset of 60 min × 24 h × 6 days = 8640 min-scale locomotor counts for each animal for each week (total of 14 weeks). Removal of the transient effect of outside disturbances to the cage is also important to evaluate the stability of locomotor dynamics. Data for one rat was found to be incomplete (owing to battery failure of devices) while two rats had uncharacteristically low locomotor counts for several periods; these rats were excluded from the analysis, giving a final total of 21 rats used. It should also be noted that the period ER_wk3_ contained data from 5 days owing to a change in the cage cleaning schedule.

#### (iv) Local statistics for locomotor activity distributions

Mean, variance and skewness of locomotor activity were calculated for each 6 day period of locomotor activity data. These statistical moments have been shown to appropriately characterize locomotor activity, particularly intermittency in locomotor dynamics, with rarer bursts of higher activity separated by longer periods with less activity, and consequently, behavioural alterations with symptomatic changes in humans [16,17].

#### (v) Cumulative distributions of resting periods

We estimated the cumulative probability distribution *P*_c_ (*T* ≥ *a*) of durations *a* (min) of resting periods during which locomotor activity levels were continuously lower than, or equal to a certain threshold [18,19]. As the choice of threshold values affects the defined durations of resting periods, various threshold values were examined for the effect of these values on the distribution parameters. We examined fixed-level thresholds (0, 1, 10 activity counts) and an adaptive threshold, the ‘non-zero mean’, introduced in previous studies [18,19]. The ‘non-zero-mean’ is the mean of the activity level excluding an activity level of 0, calculated for each rat and each experimental period. As a result, in this study, the non-zero mean was found to be the threshold, giving rise to the best linearity of the log *P*_c_ (*T* ≥ *a*) − log *a* plot that ensures the robust parameter estimation explained below (see equation (2.4)) as is expected from previous studies [18,19]. Cumulative probability distributions were obtained by numerically integrating the probability density function *p*(*t*) estimated from the whole recording period with a bin width of 1 min (definable as discrete distributions taking integer values given bin width) as follows:2.3
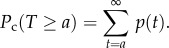


Following our previous work [18,19], a power-law form:2.4
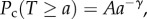
was fitted to the cumulative distributions of resting periods on the basis of a linear least-squares method using the log-transformed variables, log(*P*_c_) and log(*a*). We set the fitting range to 1–10 min. Smaller (larger) values for *γ* imply higher (lower) levels of the intermittency described above.

#### (vi) Time–frequency analysis using continuous wavelet transform

Time–frequency analysis was used to study circadian and ultradian rhythms in locomotor activity over the different experimental periods as has been done elsewhere [20]. The continuous wavelet transform (CWT) was used to decompose the locomotor time series into the time and frequency domains [21]. The CWT of a signal *x*(*t*) is the convolution of the signal with a scaled and translocated basis function called the mother wavelet *ψ*(*t*):2.5
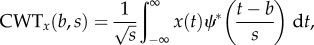
where * indicates the complex conjugate. The translocation parameter *b* focuses on a particular part of the signal in time for analysis, while the scaling parameter *s* changes the frequency content of the mother wavelet. Small values of *s* correspond to higher frequencies, while large values of *s* correspond to lower frequencies. The Morlet wavelet (which is a sinusoidal wave-based wavelet suitable for examining rhythmicity) with the following form was used as the mother wavelet:2.6
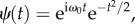
with a non-dimensional frequency of *ω*_0_ = 5 [22]. This form is chosen for its fast-decaying oscillating waveform in the time domain and compactness in the frequency domain, which enables efficient extraction of periodic patterns (i.e. circadian and ultradian signals), making it suitable for analysis of animal locomotor activity [21]. The relationship between scale *s* and frequency *f* is given by2.7
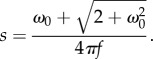


In this study, we calculated the wavelet transform from 1 to 720 cycles d^−1^ (i.e. 1 cycle every 2 min). To obtain the spectrum of locomotor activity, we calculated the power of the wavelet coefficient defined by2.8
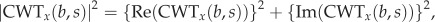
where Re(CWT*_x_*(*b*, *s*)) and Im(CWT*_x_*(*b*, *s*)) are the real and imaginary parts of the wavelet transform, respectively. Relative ultradian band power was calculated by2.9
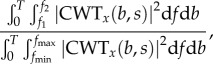
using the relationship between *s* and *f* described above, with *T* = 6 days, *f*_min_ = 1 cycle d^−1^, *f*_max_ = 720 cycles d^−1^, *f*_1_ = 2 cycles d^−1^ and *f*_2_ = 5 cycles d^−1^. Circadian power was defined as2.10
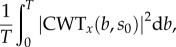
where *s*_0_ is the time scale corresponding to circadian frequency, i.e. *f* = 1 cycle d^−1^, calculated with equation (2.7).

#### (vii) Phase space reconstruction

Single-dimensional analysis, which is often used for analysing critical transitions, may not be sufficient for analysing the locomotor activity in this study because the activity exhibits oscillatory behaviour; i.e. circadian oscillations which cannot be adequately reconstructed in one-dimensional phase space. To examine the rhythmicity and consistency of circadian rhythms for the different experimental periods of interest, we reconstructed two-dimensional trajectories (limit cycles) by embedding the locomotor activity time series using a delayed coordinate [23]. Then, the embedded two-dimensional time series was used in the (two-dimensional) entropy analysis to measure the level of disorganization of circadian oscillations. Data were first pre-processed using a moving average:2.11
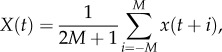
where *x*(*t*) denotes the original time series and *X* denotes the data after the moving average is applied. A smoothing parameter *M* = 120 (min) was used yielding 8400 points of data per experimental period (except for ER_wk3_ which contained 6960 points). After applying the moving average, we introduced the two-dimensional variable (*X*, *Y*) as *X* = *X*(*t*), *Y* *=* *X*(*t−τ*) (*τ* = 360 min).

Here, the delay time *τ* was defined as a quarter (6 h) of the oscillation period of the circadian rhythm (1 day). Among some proposed methods to determine delay time, such as the first zero crossing of the autocorrelation and the first minimum of the mutual information, we chose this quarter criterion (24 h/4), which is simple but reliable when the period of the basic oscillation is known, as in the current case (circadian rhythm: 24 h) [24]. When the chosen criterion is appropriate, the limit cycle appears circular; elliptical or linear limit cycles would indicate unsuitable criteria.

We calculated two-dimensional probability distribution maps *P*(*X_i_*,*Y_j_*) with a bin size of 1 count min^−1^ × 1 count min^−1^, based on which the two-dimensional entropy was calculated:2.12
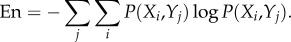


#### (viii) Statistics

**Figure 1.**
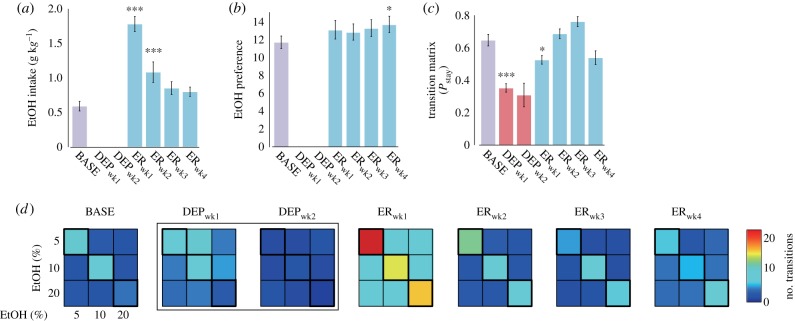
Drinking behaviour. (*a*) Ethanol (EtOH) consumption, (*b*) EtOH preference and (*c*) stay ratio. (*d*) Transition matrices showing transitions between alcoholic solutions for experimental periods. Error bars show s.e.m., N.B. no alcohol was in bottles during DEP_wk1_ and DEP_wk2_. DEP_wk2_ had extremely low numbers of accesses; stay ratio is for illustrative purposes only. ***p < 0.001; *p < 0.05.

**Figure 2.**
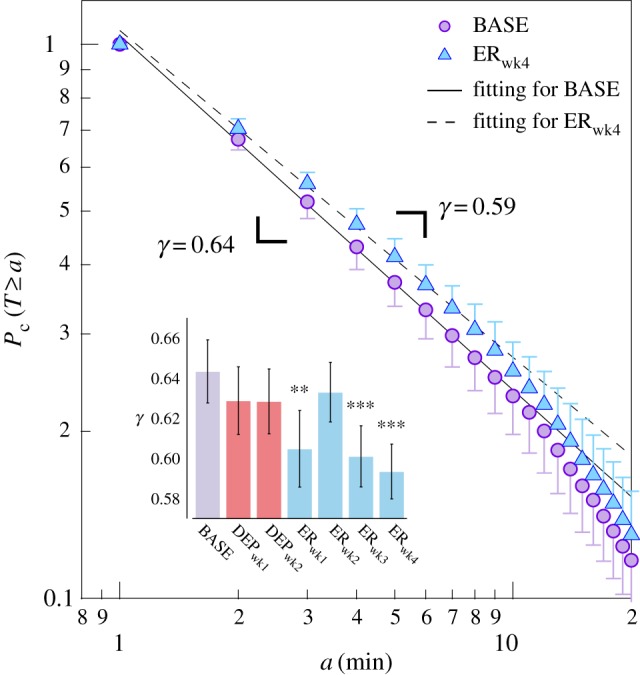
Intermittency in locomotor activity patterns. Cumulative probability distributions (discrete distributions taking integer values, given bin widths of 1 min) of resting periods in BASE and ER_wk4_, where *γ* is the slope. Inset shows decreases in scaling exponent *γ* over experimental periods indicating increased resting. We set the final fitting range to 1–10 min, and the data greater than this range did not affect the calculation of scaling exponent *γ*. Probability distribution error bars show s.d. (one-side shown only for visibility). Error bars on inset show s.e.m. ***p < 0.001; **p < 0.01.

**Figure 3.**
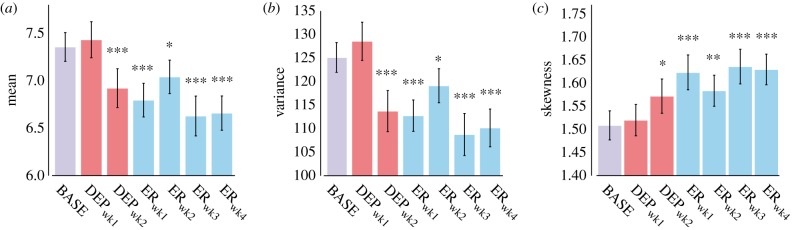
Statistical moments of locomotor activity. Decreased mean (*a*) and variance (*b*) accompanied by increased skewness (*c*) suggestive of increased intermittency. Error bars show s.e.m. ***p < 0.001; **p < 0.01; *p < 0.05.

**Figure 4.**
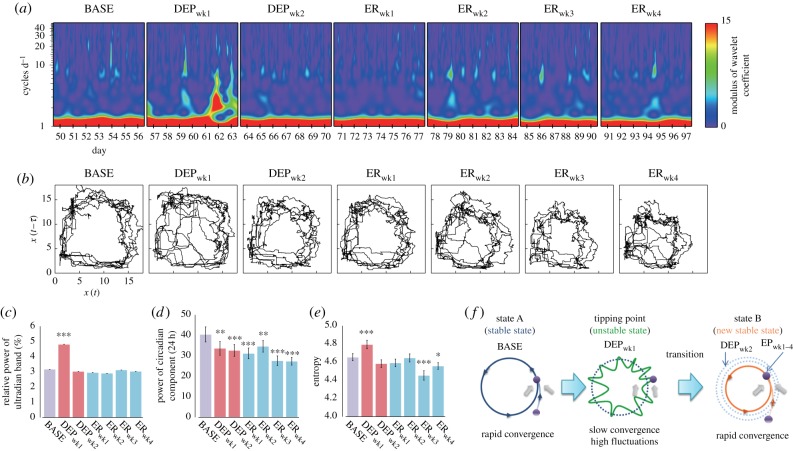
Instability during DEP_wk1_ bridges two stable states. (*a*) Wavelet time–frequency analysis and (*b*) phase space plots showing circadian trajectories for a representative animal over experimental periods. Group average (*c*) relative ultradian power, (*d*) circadian band power, and (*e*) two-dimensional entropy of the phase space plots. (*f*) Schematic illustrating limit-cycle trajectories in different states. Error bars show s.e.m. ***p < 0.001; **p < 0.01; *p < 0.05.

One-way repeated-measures analysis of variance (rmANOVA) was used to analyse differences across experimental periods for: ethanol consumption, ethanol preference, transition matrix indices, local statistics for locomotor data, activity-rest parameters, wavelet band power data and two-dimensional entropy data. For measures which were indices of preference (ethanol consumption, ethanol preference), DEP_wk1_ and DEP_wk2_ were not included because bottles contained no alcohol. For all other measures, all experimental periods were analysed. When significant effects of experimental period were found, we compared all periods using Tukey's tests; although differences between BASE and other periods were expected, differences across other periods were also of interest. In bar graphs (figures 1*a–c*, 2, 3*a–c* and 4*c–e*; electronic supplementary material, figure S5), comparisons versus baseline are indicated. Statistical analyses were performed in R v. 3.2.5. [25] (see the electronic supplementary material for additional details).

## 3. Results

With the reintroduction of ethanol solutions after deprivation, an increase in ethanol consumption (figure 1*a*) and preference for stronger alcohol typical of the ADE ensued [11] (figure 1*b*). rmANOVA revealed a main effect of experimental period on ethanol consumption (*F*_4,80_ = 41.16, *p* < 0.001) and preference (*F*_4,80_ = 2.96, *p* < 0.05). Post hoc comparisons showed that consumption was highest in ER_wk1_ compared with all other periods (*p* < 0.001) and also higher in ER_wk2_ compared with BASE (*p* < 0.001). Preference for stronger ethanol solutions increased over time (see the electronic supplementary material, figure S2), with the difference significantly higher than BASE in ER_wk4_ (*p* < 0.05) and at the trend level in ER_wk3_ (*p* = 0.074). Although preference was not significantly higher than BASE in ER_wk1_ (*p* = 0.119), this is in part owing to the ADE itself; upon reintroduction, rats accessed all bottles at an increased rate.

Drinking behaviour was also analysed by calculating stay ratios (figure 1*c*) and computing state-transition matrices (figure 1*d*; electronic supplementary material, table S1). A significant main effect of experimental period was found on the stay ratio (*F*_5,120_ = 24.18, *p* < 0.001). Post hoc analyses revealed significantly higher probabilities of staying with the same bottle during BASE and ER_wk1_–ER_wk4_ than DEP_wk1_ (*p* < 0.001 for all). Descriptively, during BASE, rats tended to stay with the same alcoholic solution, switching to other solutions at low rates (diagonal squares, figure 1*d*). During DEP_wk1_, increased switching was observed in bottles where alcoholic solutions had been held, suggesting the occurrence of alcohol-seeking/craving. In DEP_wk2_, the greatly decreased number of transitions is consistent with animals giving up alcohol-seeking after continuously being offered only water. Upon ethanol reintroduction (ER_wk1_), there was a greatly increased amount of alcohol consumed (the ADE) while staying behaviour re-emerged and progressively re-stabilized with fewer switches during ER_wk2_, ER_wk3_ and ER_wk4_, (diagonal squares, figure 1*d*) accompanied by a decreased number of stays with weak solutions (5%) and an increased number of stays with stronger solutions (20%) (electronic supplementary material, table S1). Consumption of higher-concentration alcohol solutions (20%) following deprivation leads to a more rapid rise in blood alcohol concentrations and subsequent intoxication [11].

### (a) Increased intermittency in locomotor activity patterns also indicate a state transition

Following ethanol reintroduction, statistical and dynamical characteristics of locomotor activity underwent significant changes suggestive of increased intermittency, with rarer bursts of higher activity separated by longer periods with less activity. To evaluate alterations in dynamical intermittency, cumulative probability distributions of ‘resting periods’ (continuous periods below a non-zero mean threshold) of locomotor activity were analysed [18,19]. Figure 2 shows the group mean cumulative probability distributions for BASE and ER_wk4_, of which the power-law exponent *γ* is the absolute slope (see Material and methods). Decreases in *γ* were found over experimental periods, with a significant main effect of the experimental period (figure 2, inset) (*F*_6,120_ = 7.36, *p* < 0.001). Post hoc comparisons showed that *γ* was significantly lower in ER_wk1_ (*p* < 0.01), ER_wk3_ and ER_wk4_ (both *p* < 0.001) than in BASE, suggesting systematically more episodes of longer rest following deprivation.

These results were also supported by changes observed in statistical moments (mean, variance, skewness) [16,17] of locomotor activity, suggestive of increased intermittency as experimental periods progressed (figure 3*a–c*). Lower mean and variance were indicative of long periods with less activity, and higher skewness indicated bursts of increased activity. Main effects of experimental period were found on the mean (*F*_6,120_ = 23.72, *p* < 0.001), variance (*F*_6,120_ = 28.99, *p* < 0.001) and skewness (*F*_6,120_ = 12.76, *p* < 0.001). Post hoc tests indicated that compared with BASE, the mean was significantly decreased in DEP_wk2_, ER_wk1_, ER_wk3_, ER_wk4_ (*p* < 0.001 for all) and ER_wk2_ (*p* < 0.05). Variance was lower in ER_wk1_, DEP_wk2_, ER_wk3_, ER_wk4_ (*p* < 0.001 for all) and ER_wk2_ (*p* < 0.05) than in BASE. Skewness was significantly higher in DEP_wk2_ (*p* < 0.05), ER_wk1_, ER_wk3_, ER_wk4_ (*p* < 0.001 for all) and ER_wk2_ (*p* < 0.01) compared with BASE.

Together, the analysis of cumulative probability distributions of ‘resting periods’ and other statistical moments suggests that after the first week of deprivation, rats transitioned into a new allostatic state with increased intermittency.

### (b) Identifying early-warning signals during deprivation

Statistical properties and rhythmicity of locomotor activity data were further examined, finding that circadian and ultradian patterns underwent a dynamical state transition after the first week of deprivation (figure 4*a–e*). Figure 4*a* shows results of the wavelet analysis for a single representative rat (electronic supplementary material, figure S3 contains plots for all rats). Increases in diurnal relative wavelet power were present during DEP_wk1_, suggesting increased ultradian rhythms (figure 4*c*). A significant main effect of the experimental period (*F*_6,120_ = 21.39, *p* < 0.001) was found and post hoc comparisons showed that the relative wavelet power during DEP_wk1_ was significantly higher than in all other periods (*p* < 0.001). The circadian power was also analysed for differences, finding a main effect of the experimental period (*F*_6,120_ = 15.38, *p* < 0.001) (figure 4*d*). Post hoc comparisons showed that the power was significantly larger for BASE than in DEP_wk1_ (*p* < 0.01), DEP_wk2_ (*p* < 0.001), ER_wk1_ (*p* < 0.001), ER_wk2_ (*p* < 0.01), ER_wk3_ (*p* < 0.001) and ER_wk4_ (*p* < 0.001) with a sequentially decreasing pattern.

Circadian dynamics of locomotor activity were reconstructed into two-dimensional limit cycles (figure 4*b*; electronic supplementary material, figure S4, left) yielding two-dimensional probability density diagrams of their trajectories (electronic supplementary material, figure S4, right). The circular limit cycles which were observed indicated that the quarter criterion used was suitable and that the phenomenon of slowing down was well extracted. The limit cycles showed changes across experimental periods, starting large (in terms of circadian power, figure 4*d*) and stable during BASE, losing the clear cycle (becoming diffuse and disorganized) during DEP_wk1_, and retaking a more compact (figure 4*d*) and stable limit cycle during DEP_wk2_ and the ER weeks. We also calculated the moment of inertia about the centre of mass (average squared radius, electronic supplementary material, figure S5) for limit cycles, finding that the average squared radius is significantly larger in BASE than all other periods after DEP_wk1_. The two-dimensional entropy values were examined to measure the disorder of this circadian organization and a significant main effect of experimental period was found (*F*_6,120_ = 21.62, *p* < 0.001) (figure 4*e*). Post hoc comparisons showed that two-dimensional entropy was significantly higher for DEP_wk1_ than for all other periods (all *p* < 0.001), suggesting increased instability. These results reflect a dynamical transition from a large, stable circadian oscillation to a small, stable one (figure 4*d* for the circadian power; electronic supplementary material, figure S5 for the squared radius for limit cycles; figure 4*f* for schematic) preceded by instability of circadian rhythms (figure 4*e*) and a resultant increase in ultradian power (i.e. slowing down; figure 4*d*), serving as early-warning signals during the first week of deprivation.

## 4. Discussion

Here, we acquired ILD sets of drinking behaviour and locomotor activity in a rat model of alcohol addiction and used a multi-scale computational approach to demonstrate a critical transition from controlled baseline alcohol consumption to excessive alcohol drinking (figure 4*f*). This state transition is preceded by a critical slowing down scenario (an early-warning signal) during early deprivation. We propose that our approach has the potential to make important contributions to the understanding of disease onset in general and can be translated to the clinical situation by the appropriate use of wearable and mobile biomedical sensing technology [4].

The theory of critical transitions has recently attracted interest not only in a broad range of scientific fields [5,6], but also in the field of clinical medicine, with the expectation that its concepts will contribute to a detailed understanding of disease onset and progression [3]. In particular, the presence of early-warning signals such as critical slowing down has the potential to enable prediction of transitions to or between pathological states. There are studies suggesting the occurrence of critical transitions in clinical contexts [26,27], but these investigations used snapshots of systems at separate time points exhibiting stable and unstable states near bifurcation points. While these studies used models to simulate the state-transition phenomenon, it has been pointed out that the existence of abrupt, disease-related and contiguous state transitions has not been empirically proven [28,29]. Hence, the analysis presented here is evidence in support of the hypothesis of a disease-related dynamical state transition.

Through the course of the baseline period, rats acquired some level of preference for lower percentage alcohol solutions (5 and 10%); like humans, they take some time to get accustomed to the taste of alcohol. This is consistent with the literature—rats initially prefer these weaker alcohol solutions owing to the slightly sweet taste, but find the smell and taste of strong (20%) solutions aversive [11]. Previous studies have shown that without a deprivation period, or with a too short deprivation period, excessive relapse-like drinking with a shift of preference to stronger alcohol is not observed [11]. Shifts are only observed with protracted periods of deprivation, suggesting that changes are not gradual but abrupt.

During the final week of the baseline period, transition matrices show that rats had a strong tendency to stay with the same solution during successive accesses. By contrast, the first week of deprivation was characterized by a marked increase in switching between bottles which is consistent with alcohol-seeking behaviour during deprivation; i.e. craving for alcohol. Following the reintroduction of alcohol solutions, there was a return of an increasingly strong stay ratio with one important difference, an increased preference for stronger alcohol (20%), suggesting that the rats settled into a new, stable, allostatic state. An allostatic model for alcoholism has been proposed, integrating molecular, cellular and circuitry neuroadaptations in brain motivational systems produced by chronic alcohol consumption [30,31]—we now provide quantitative empirical evidence that allostatic switches occur following long-term voluntary alcohol consumption and deprivation.

The sudden behavioural shift observed in drinking patterns was also demonstrated in both the statistics describing local dynamics (figure 3*a–c*) and the circadian organization of locomotor activity (figure 4*c–e*). Properties found in various multi-scale complex systems can be observed in the local dynamics of intensive longitudinal locomotor activity time series [2,18,19], and the statistical properties of these data are shown to be related to pathological states in depression [18,32], schizophrenia [32,33] and bipolar disorder [2,34]. Particularly, it has been observed that disease states can be characterized by increased intermittency in locomotor activity [17]. The degree of intermittency was evaluated using two different approaches. First, intermittency in locomotor activity has been shown to be effectively captured by statistical moments and particularly a combination of mean and skewness [16,17]. The decreased mean and variance across experimental periods indicate decreased overall activity, while the increased skewness reflects the presence of occasional or intermittent bursts. Specifically, mean, variance and skewness after deprivation are significantly different in these directions, suggesting that a transition between two states is also seen in local locomotor dynamics.

Second, the dynamical aspects of intermittency were examined by analysing probability distributions of resting periods in rest–activity transitions. Decreases in the scaling exponent *γ* indicate increased intermittency in locomotor activity, or systematically higher probabilities of observing longer resting periods [18], tendencies which were observed after rats transitioned into a new allostatic state. These changes are consistent with those previously observed in patients with depression [18], schizophrenia [33] and bipolar disorder [2], illnesses known to show high comorbidity with alcohol-use disorder [35]. Increased intermittency in locomotor activity has been also observed in mice with circadian clock gene (*Per2*) mutations [19,20]. These mutant mice also exhibit increased preference for and consumption of alcohol [36]. Furthermore, a theoretical model for intermittent behavioural dynamics and its alterations has been proposed [20] based on a priority stochastic queuing theory [37]. This model explains increased intermittency as strategic changes in decision-making to initiate actions with preferential selectivity to demands with higher priority (in this case, alcohol), showing tendencies to react only to higher demands (generating occasional activity bursts) while remaining quiet most of the time resulting in reduced activity. Within the framework of this model, the changes observed here in locomotor activity may reflect enhanced vulnerability towards alcohol and may thus mark a transition towards addictive behaviour.

It is of note that changes seem to start in the middle of the deprivation period (DEP_wk2_), suggesting that the state transition is spontaneous in nature. This finding is in line with a recent cross-sectional study [10] which suggests a dopaminergic mechanism behind the differences between the first and second weeks of deprivation. This study observed a time-dependent effect during deprivation, finding a hypo-dopaminergic state during the first week and a hyper-dopaminergic state after the first week. The abrupt changes we observed in our longitudinal dataset were on a similar timeline and appear to reflect neural changes and adaptations in regulatory reward circuitry. Our results also point to early deprivation (the first of two weeks) as a critical driver for the onset of addictive behaviour which is consistent with the literature on alcohol addiction emphasizing the importance of deprivation in the development of addiction [8,11].

It has been shown that alcoholic patients experiencing alcohol withdrawal show disrupted sleep [38] and melatonin release [39], while in rodents, long-term alcohol intake and alcohol withdrawal have also been shown to affect circadian rhythms of drinking activity [40,41] and free wheel running activity [42,43]. In particular, in animals exposed to up to 20% alcohol solutions (but not when exposed to up to 10%), decreased circadian amplitudes were found during alcohol deprivation [40,44]. In this study, we used continuous measurements of locomotor activity, finding decreased circadian power during both deprivation and reintroduction phases (figure 4*d*). Moreover, decreases in circadian power began when the deprivation started and not before even though rats had been consuming alcohol for eight weeks. This finding is important as it suggests that it is not alcohol consumption that causes disruptions in circadian rhythms *per se*, but instead the deprivation that may be the culprit.

Studies on critical transitions have identified the presence of early-warning signs of abrupt transitions or shifts from one state to another [5,6]. The most well known of these is ‘critical slowing down’, i.e. the appearance of low-frequency, highly correlated behaviour near bifurcation, or tipping points where there is low resilience to perturbations owing to system instability [5,6]. In this study, we found increased instability of circadian locomotor rhythms and increased ultradian rhythms by using wavelet analysis (i.e. multiple cycles per day, figure 4*a*), specifically during the first week of deprivation. This suggests that early deprivation is characterized by dynamics often seen when systems are close to bifurcation points, and the increase in ultradian rhythms can be regarded as an early warning of a transition into excessive relapse-like drinking via the critical slowing down scenario. Moreover, we observed alterations in the dynamical signatures of stability of attractors (limit cycles) themselves in phase space. More disorganized and dynamically unstable trajectories were observed during the first week of deprivation, signalling a transition from large stable limit cycles during baseline to smaller stable limit cycles during protracted deprivation and subsequent excessive drinking periods (illustrated by figure 4*f*).

The potential indicators for early warning identified in the literature so far present analytical techniques, such as using autocorrelation coefficients at a specific scale [5], which can be augmented [3,6] by using a multi-dimensional and multi-scale approach such as the current one. This method clearly captures transitions between different states, differentiating not only between stability and instability but also characterizing the distinct stable baseline and vulnerable state for addicted behaviours themselves. Importantly, using this approach on contiguous and longitudinal data enables visualization of transitions as they happen.

It might be thought that excessive drinking and the increase in preference observed is the natural progression when rats are given prolonged continuous exposure to alcohol. However, as seen in the electronic supplementary material, figure S2, this is not the case—during BASE, drinking behaviour is already stable and the deprivation is what drives the system to instability, after which it transitions into the new steady state that is characterized by excessive alcohol consumption, especially of higher concentrated alcohol solutions.

It should be recognized that the ADE model and alcohol deprivation represent just a single model of disease onset. Other types of disease are characterized by gradual change in response to stressors over time (i.e. gradual deterioration of mental state) and may follow alternative courses. As the present model involves an external event pushing the system to a state of instability, followed in time by a transition, the approach used here is likely to have applications in psychiatric disorders which are phasic or episodic in nature that are often externally triggered (e.g. substance use disorders, major depression, bipolar disorder, post-traumatic stress disorder). The next step is to verify, translate and adapt these methods in other disease scenarios.

Thus, the results and especially the computational methods applied here are considered to outline an adaptable framework for processing ILD and understanding disease dynamics in the biomedical field. Indeed, the initial idea of ‘dynamical disease’ proposed almost 40 years ago [45] discussed bifurcations between normal and pathological oscillations in a variety of illnesses. While the implementation and realization of this concept have been difficult [2], the use of biomedical ILD to examine limit-cycle bifurcations may now begin to substantiate the concept of dynamical disease using the untapped potential of sensing and measuring technologies which have enabled the non-invasive acquisition of longitudinal and contiguous data from various behavioural and physiological patterns. Also, the ability to identify early-warning signals which precede disease onset, together with prospective translational studies, will lead to the timely implementation of preventive measures and treatments, and provide far-reaching ICT applications in healthcare [2,4].

## Supplementary Material

Figure S1; Figure S2; Figure S3; Figure S4; Figure S5

## Data accessibility

Drinking behaviour and locomotor activity data have been uploaded to Dryad (http://dx.doi.org/10.5061/dryad.t3t7p) [46].
