# Supplementary material for: Dynamical state transitions into addictive behaviour and their early-warning signals
Source: Proc Biol Sci. 2017 Aug 2;284(1860):20170882. doi: 10.1098/rspb.2017.0882 (PMC5563804; doi:10.1098/rspb.2017.0882)
Supplement: Figure S1; Figure S2; Figure S3; Figure S4; Figure S5 [file rspb20170882supp1.pdf]

Supplementary Materials

Additional detail for statistics

Statistical analyses to compare the effects of experimental periods were performed in R 3.2.5. (R Core Team (2016). R: A language and environment for statistical computing. R Foundation for Statistical Computing, Vienna, Austria. URL https://www.R-project.org/)

One-way repeated measures analysis of variance (rmANOVA) was used to examine the differences across experimental periods given the repeated nature of measurements. rmANOVA models were specified using the lme function of the 'nlme' package (Pinheiro J, Bates D, DebRoy S, Sarkar D and R Core Team (2016). _nlme: Linear and Nonlinear Mixed Effects Models_. R package version 3.1-128). Models were specified to examine the effect of experimental period (the within-subjects factor) on the dependent variables of interest: ethanol consumption, ethanol preference, transition matrix indices (P_{stay}), local statistics for locomotor data (mean, skewness, variance), activity-rest parameters(γ), wavelet band power data (circadian and relative ultradian) and 2D entropy data. Rats were specified as subjects, and a random statement where periods were nested in rats was included. For ethanol consumption and ethanol preference, DEP_{wk1} and DEP_{wk2} were not included because bottles contained no alcohol. For P_{stay}, DEP_{wk1} was included for comparison of stability (while DEP_{wk2} was also calculated, a low number of accesses/transitions, [e.g. no accesses or only single accesses during the week] resulted in the index being uninformative- we refrain from making any interpretation). For all other measures, all experimental periods were analysed. (To confirm that the assumption of sphericity was not violated, we used the ezANOVA function of the 'ez' package).

For all models, whenever significant effects of experimental period were found by the rmANOVA (significance level of p <0.05), it was necessary to identify where the differences lay. Although we were generally interested in comparison of BASE to other periods, these differences were already expected given prior knowledge about the paradigm. As such, post-hoc multiple comparisons were performed with the Tukey contrasts to search for differences across all periods. Tukey contrasts were calculated using the glht function of the 'multcomp' package (Torsten Hothorn, Frank Bretz and Peter Westfall (2008). Simultaneous Inference in General Parametric Models. Biometrical Journal 50(3), 346--363).

Figure S1

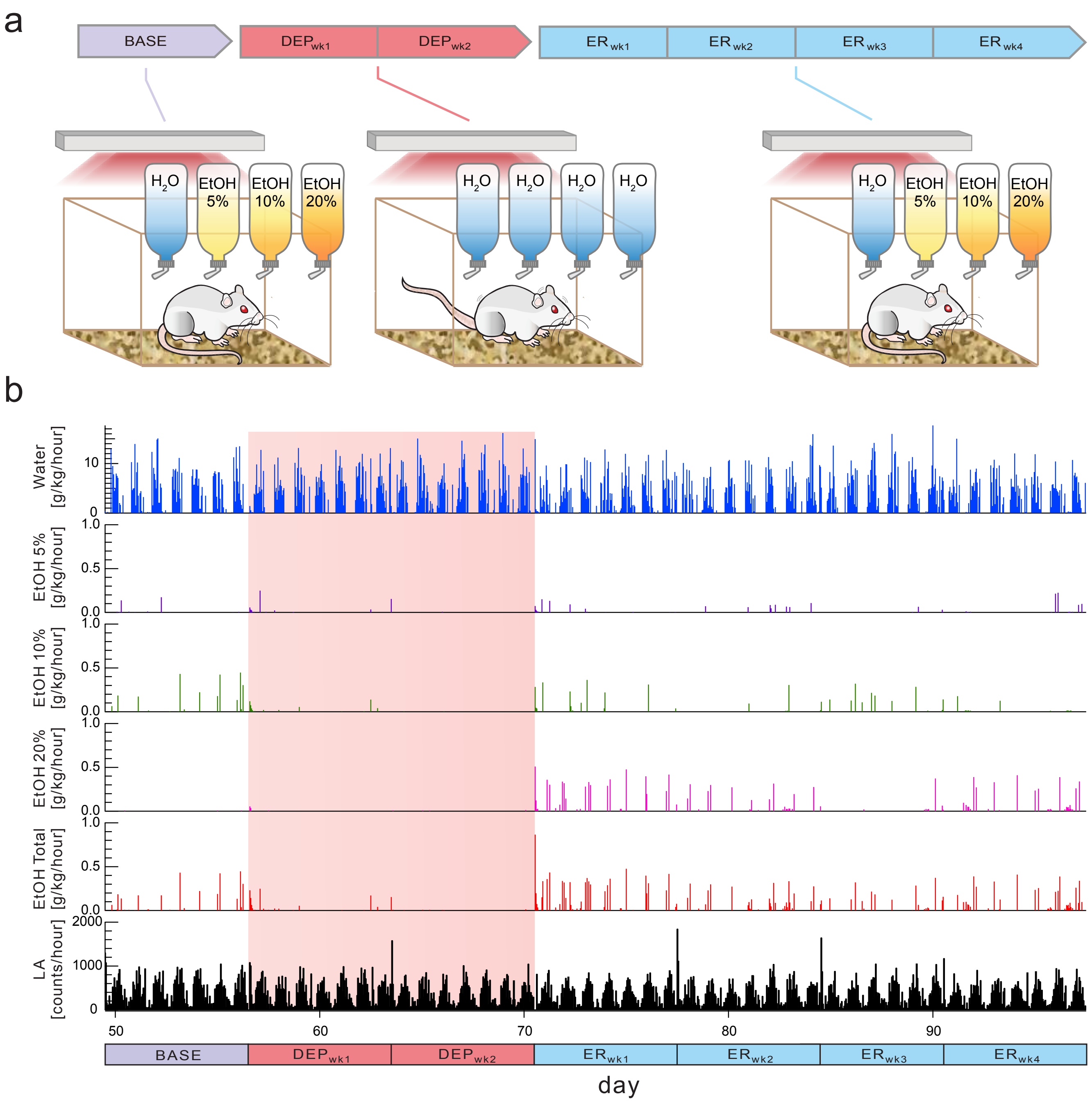
a
BASE
DEPwk1
DEPwk2
ERwk1
ERwk2
ERwk3
ERwk4
H2O
EtOH 5%
EtOH 10%
EtOH 20%
b
Water [g/kg/hour]
EtOH 5% [g/kg/hour]
EtOH 10% [g/kg/hour]
EtOH 20% [g/kg/hour]
EtOH Total [g/kg/hour]
LA [counts/hour]
day

Figure S1. Alcohol deprivation effect (ADE) paradigm and sample intensive longitudinal data (ILD). **(a)** Schematic of the ADE paradigm: baseline (BASE) followed by two weeks of deprivation (DEP_{wk1} and DEP_{wk2}) and four weeks of ethanol (EtOH) reintroduction ($ER_{wk1\text{-}4}$), and time schedule. **(b)** Drinking (Water, 5%, 10%, 20%, total EtOH) and locomotor activity (LA) traces for a sample rat during experimental periods as an example of ILD. *NB*: During deprivation (red shaded area) all bottles contained water only; accesses shown then are water and not EtOH.

Figure S2

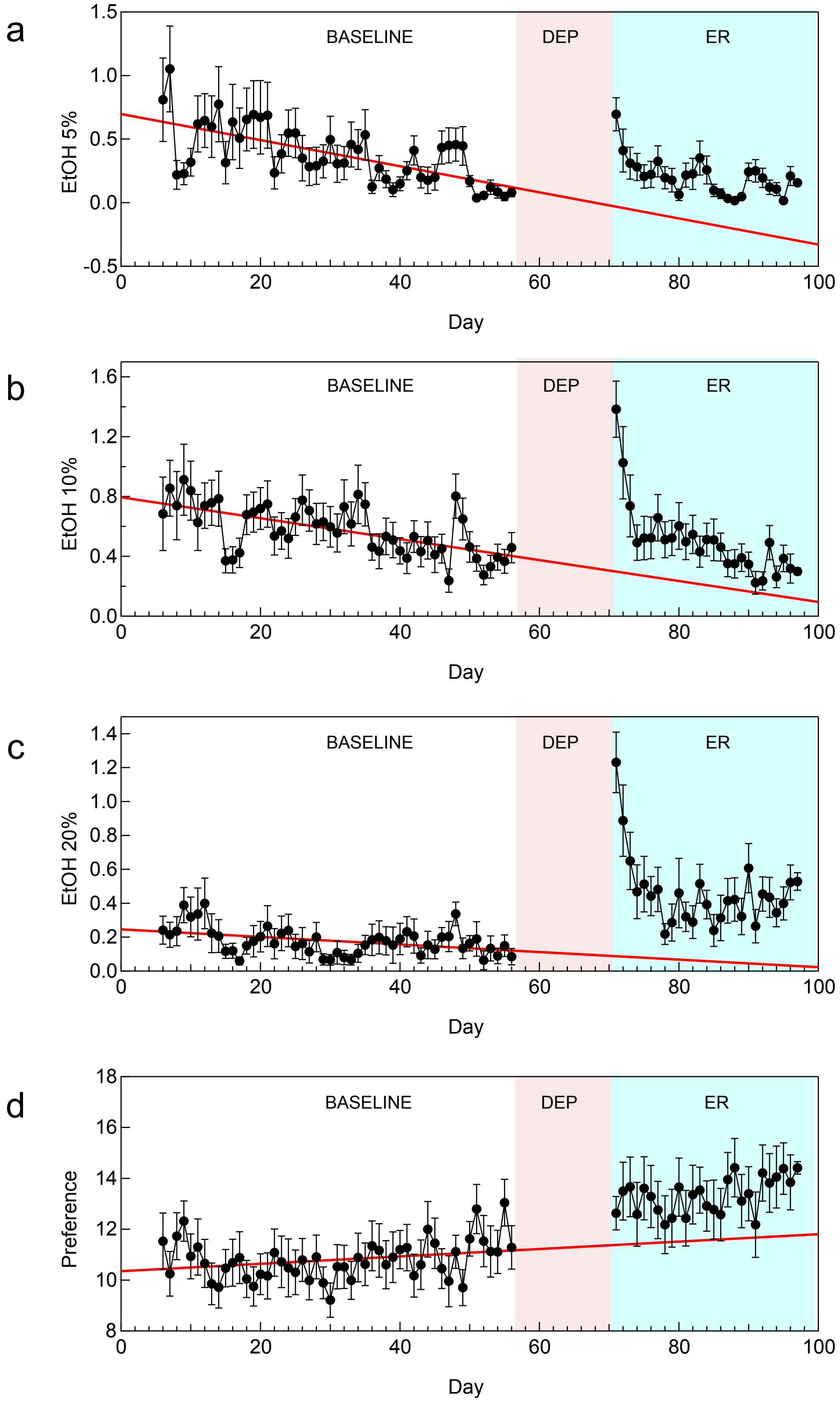
a
b
c
d
BASELINE
DEP
ER
EtOH 5%
EtOH 10%
EtOH 20%
Preference
Day
1.5
1.0
0.5
0.0
-0.5
1.6
1.2
0.8
0.4
0.0
1.4
1.2
1.0
0.8
0.6
0.4
0.2
0.0
18
16
14
12
10
8
0
20
40
60
80
100

Figure S2. Average daily consumption of a) 5%, b) 10%, and c) 20% solutions, and d) average preference during the course of the experiment, demonstrating the alcohol deprivation effect. Regression lines showing projected trajectories and were fitted using the curves for averages from day 5-56 (until deprivation). Error bars denote standard deviation. Data from days 1-5 was lost due to sensor calibration issues.

Figure S3

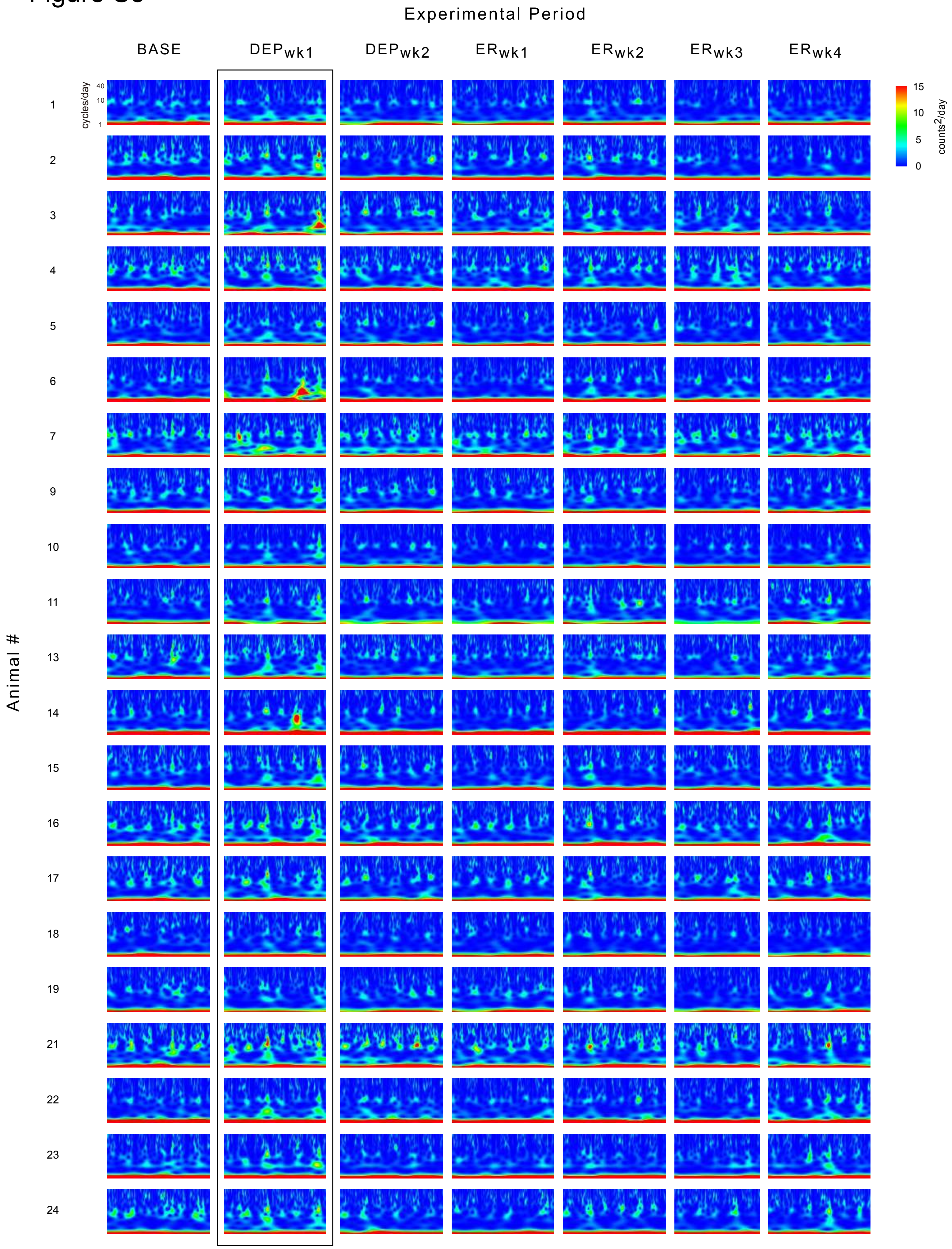
Experimental Period
BASE
DEPwk1
DEPwk2
ERwk1
ERwk2
ERwk3
ERwk4
Animal #
1
2
3
4
5
6
7
9
10
11
13
14
15
16
17
18
19
21
22
23
24
cycles/day
40
10
1
15
10
5
0
counts²/day

Figure S3. Wavelet plots for all animals showing the distribution of power of the locomotor activity signal across different frequencies and how it changed over time (experimental periods)**.** Values of moduli of the wavelet coefficients (powers) are colour coded according to their magnitude (blue indicates a low and red a large value), and the ordinates are represented on a logarithmic scale. Increased power at low frequencies was observed in DEP_{wk1}, showing instability in ultradian rhythms and suggestive of the system being near a tipping point.

Figure S4

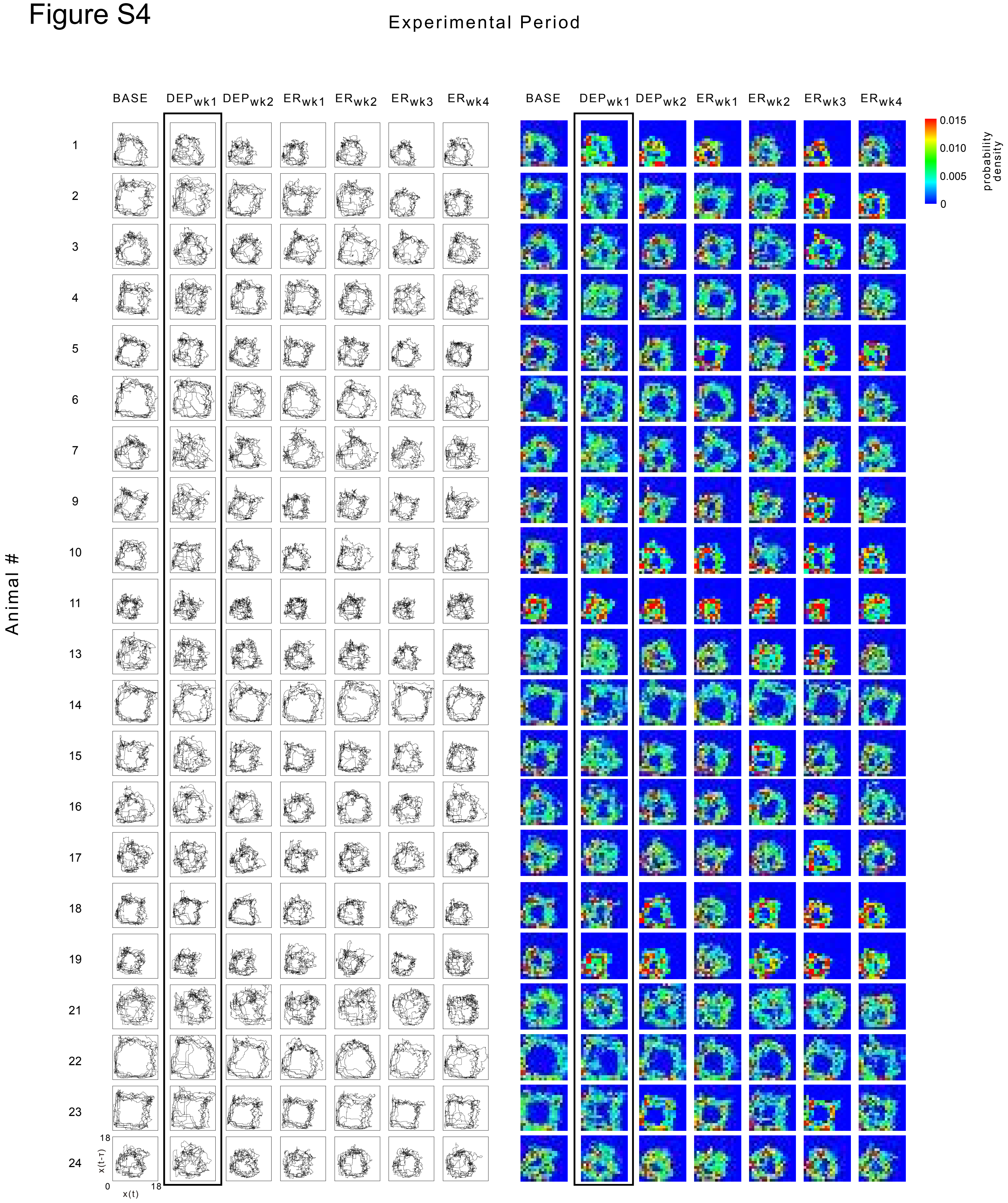
Experimental Period
BASE
DEPwk1
DEPwk2
ERwk1
ERwk2
ERwk3
ERwk4
Animal #
1
2
3
4
5
6
7
9
10
11
13
14
15
16
17
18
19
21
22
23
24
18
0
18
x(t)
x(t-τ)
0.015
0.010
0.005
0
probability density

Figure S4. Limit cycle trajectories (left) and probability density maps (right) of rat locomotor activity over experimental periods**.** In BASE, plots show a large clear circle indicating stable circadian rhythms. In DEP_{wk1} trajectories become diffuse suggesting instability. From circles subsequently decrease in size, and once again become clear circles, suggestive of a stabilisation into a new state (DEP_{wk2} – ER_{wk4}).

Figure S5

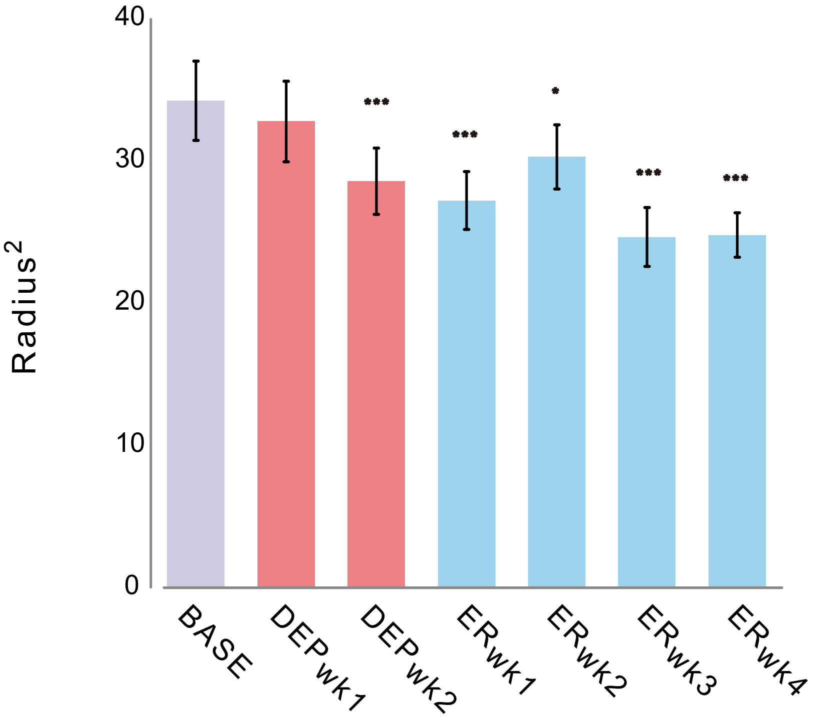

40
30
20
10
0
Radius^2
***
***
*
***
***
BASE
DEPwk1
DEPwk2
ERwk1
ERwk2
ERwk3
ERwk4

Figure S5. Moment of inertia about centre of mass (average squared radius) for limit cycles shown in figure S3 confirms the decrease in the size of the cycle across experimental periods. Note that the squared radius in DEP_{wk1} is not significantly smaller than that in BASE, different from the observation for the circadian power (figure 5*d*), presumably because this quantity include increased ultradian power in DEP_{wk1} (figure 5*c*). Error bars show S.E.M. *** $p < 0.001$; ** $p < 0.01$; * $p < 0.05$.

Changes in drinking behaviour described by transitions between solutions over experimental periods.

**Period**		**Average Transitions**			
		H_2O	5%	10%	20%
	H_2O	622.14	15.62	18.10	12.14
BASE	5%	16.86	**10.81**	2.14	1.48
	10%	17.76	1.43	**8.48**	1.67
	20%	13.19	1.95	1.43	**3.81**
	H_2O	608.57	26.14	23.52	10.00
DEP_{wk1}	5%	25.14	**8.95**	10.95	4.05
	10%	25.05	3.90	**8.38**	4.67
	20%	11.76	3.38	3.90	**2.05**
	H_2O	579.10	6.90	6.95	3.90
DEP_{wk2}	5%	7.43	**0.57**	0.57	1.43
	10%	7.86	0.71	**1.33**	0.71
	20%	4.90	1.33	0.71	**0.19***
	H_2O	474.62	25.90	22.81	27.05
ER_{wk1}	5%	24.62	**24.38**	7.52	7.90
	10%	23.43	8.19	**15.57**	7.81
	20%	26.29	7.81	8.19	**17.24**
	H_2O	496.10	13.86	15.10	15.10
ER_{wk2}	5%	14.29	**12.81**	2.43	1.86
	10%	15.33	1.86	**9.76**	2.00
	20%	15.95	2.10	1.86	**6.57**
	H_2O	545.38	12.90	12.57	14.81
ER_{wk3}	5%	13.05	**4.67**	0.95	0.95
	10%	13.57	1.05	**6.95**	0.81
	20%	15.48	0.86	1.05	**6.14**
	H_2O	422.57	11.48	9.67	17.29
ER_{wk4}	5%	11.71	**5.95**	1.81	3.10
	10%	9.90	3.71	**5.24**	3.71
	20%	16.76	3.33	3.71	**9.76**

Table S1. Group average number of transitions per period for Water, 5%, 10% and 20% alcohol solutions. Bold numbers indicate stays with the same alcohol solution. Staying (stable) is observed in BASE with initial preference for weaker solutions, followed by increased switching during DEP_{wk1}. In DEP_{wk2} greatly decreased amounts of accesses were observed. (*During DEP_{wk2} two rats switched to the 20% bottle as their main water bottle- these accesses were interpreted as water accesses). From ER_{wk1}- ER_{wk4} staying behaviour reappeared accompanied by a shift in preference, with 20% having the most stays.
